# Association of Household Opioid Availability With Opioid Overdose

**DOI:** 10.1001/jamanetworkopen.2023.3385

**Published:** 2023-03-17

**Authors:** Michelle A. Hendricks, Sanae El Ibrahimi, Grant A. Ritter, Diana Flores, Michael A. Fischer, Roger D. Weiss, Dagan A. Wright, Scott G. Weiner

**Affiliations:** 1 Division of Research and Evaluation, Comagine Health, Portland, Oregon; 2 School of Public Health, Department of Epidemiology and Biostatistics, University of Nevada, Las Vegas; 3 Schneider Institutes for Health Policy, Heller School for Social Policy and Management, Brandeis University, Waltham, Massachusetts; 4 Section of General Internal Medicine, Boston Medical Center, Boston University School of Medicine, Boston, Massachusetts; 5 Department of Psychiatry, Harvard Medical School, Boston, Massachusetts; 6 Division of Alcohol, Drugs, and Addiction, McLean Hospital, Belmont, Massachusetts; 7 Injury and Violence Prevention Program–Public Health Division–Oregon Health Authority, Portland; 8 Department of Emergency Medicine, Brigham and Women’s Hospital, Boston, Massachusetts

## Abstract

**Importance:**

Previous studies that examined the role of household opioid prescriptions in opioid overdose risk were limited to commercial claims, did not include fatal overdoses, and had limited inclusion of household prescription characteristics. Broader research is needed to expand understanding of the risk of overdose.

**Objective:**

To assess the role of household opioid availability and other household prescription factors associated with individuals’ odds of fatal or nonfatal opioid overdose.

**Design, Setting, and Participants:**

A retrospective cohort study assessing patient outcomes from January 1, 2015, through December 31, 2018, was conducted on adults in the Oregon Comprehensive Opioid Risk Registry database in households of at least 2 members. Data analysis was performed between October 16, 2020, and January 26, 2023.

**Exposures:**

Household opioid prescription availability and household prescription characteristics.

**Main Outcomes and Measures:**

Opioid overdoses were captured from insurance claims, death records, and hospital discharge data. Household opioid prescription availability and prescription characteristics for individuals and households were modeled as 6-month cumulative time-dependent measures, updated monthly. To assess the association between household prescription availability, household prescription characteristics, and overdose, multilevel logistic regression models were developed, adjusting for demographic, clinical, household, and prescription characteristics.

**Results:**

The sample included 1 691 856 individuals in 1 187 140 households, of which most were women (53.2%), White race (70.7%), living in metropolitan areas (75.8%), and having commercial insurance (51.8%), no Elixhauser comorbidities (69.5%), and no opioid prescription fills in the study period (57.0%). A total of 28 747 opioid overdose events were observed during the study period (0.0526 per 100 person-months). Relative to individuals without personal or household opioid fills, the odds of opioid-related overdose increased by 60% when another household member had an opioid fill in the past 6 months (adjusted odds ratio [aOR], 1.60; 95% CI, 1.54-1.66) and were highest when both the individual and another household member had opioid fills in the preceding 6 months (aOR, 6.25; 95% CI, 6.09-6.40).

**Conclusions and Relevance:**

In this cohort study of adult Oregon residents in households of at least 2 members, the findings suggest that household prescription availability is associated with increased odds of opioid overdose for others in the household, even if they do not have their own opioid prescription. These findings underscore the importance of educating patients about proper opioid disposal and the risks of household opioids.

## Key Points

**Question:**

What role do household opioid prescription availability and prescription characteristics play in an individual’s odds of opioid overdose?

**Findings:**

In this cohort study of 1 691 856 Oregon adults in 1 187 140 households, the odds of opioid-related overdose increased significantly when another household member had opioid fills in the preceding 6 months. The odds also increased when both the individual and another household member had opioid fills in the preceding 6 months.

**Meaning:**

The findings of this cohort study underscore the importance of educating individuals about the risks of keeping opioids in the household.

## Teaser

This cohort study examines the risk for opioid overdose associated with the presence of opioids within the household.

## Introduction

Although the rate of opioid prescribing has decreased in recent years,^1^ deaths involving prescription opioids are not far from their 2017 peak, with 16 416 people in the US dying from prescription drug overdose in 2020.^2^ Prescription opioids are still a common entry point into problematic opioid use, with two-thirds of individuals seeking treatment for opioid use disorder reporting that they began their opioid use with oxycodone, hydrocodone, or other prescription opioids.^3^ Use of prescription opioids does not always start with a health care encounter: more than half of individuals reporting nonprescribed use initially obtained the opioids through friends or relatives.^4^ Approximately half of opioids prescribed after surgery or to outpatients with cancer go unused; because most patients are unaware of proper opioid disposal and storage, these excess opioids often end up in an unlocked household medicine cabinet.^5,6^ These opioids might then be used by other household members without a physician’s oversight, potentially leading to greater risk of adverse outcomes.

Researchers have identified several prescription-related factors associated with overdose among individuals prescribed opioids, including higher dosages, greater number of opioid prescription fills, and coprescribing with benzodiazepines.^7,8,9^ Few studies have explored the role of opioids prescribed to household members in opioid overdose,^10,11^ and they were limited to commercial claims, did not include fatal opioid overdose outcomes, and often did not examine the role of household prescription characteristics in overdose risk. We used the Oregon Comprehensive Opioid Risk Registry, a database developed by our team that links a statewide all-payer claims database to several public health data sets,^12^ to examine the contributions of individual and household prescription factors to opioid overdose.

## Methods

### Data Sources

Our data source was the 2013-2018 Oregon voluntary All Payer All Claims Database (APCD), probabilistically linked using patient identifiers (first name, last name, and date of birth) to several public health data sets, including the Oregon prescription drug monitoring program (PDMP), Oregon Vital Records death certificates, and the Oregon hospital discharge database. To conduct the linkages, we used fastLink linkage and de-duplication software in R fastLink, version 0.5.0 (R Foundation for Statistical Computing), which was published on November 12, 2018.^12,13^ This study was approved by the Mass General Brigham Human Research Committee; patient consent requirement was waived because this study posed minimal risk to participants, and the research could not practicably be conducted without the waiver. We followed the Strengthening the Reporting of Observational Studies in Epidemiology (STROBE) reporting guideline.

The Oregon voluntary APCD includes all Medicaid patients, individuals covered by Medicare Advantage, and approximately 80% of commercially insured individuals. We excluded patients with Medicare fee-for-service plans from the linkage because of data use agreement restrictions. We used the PDMP as the data source for all prescription information. The PDMP contains all controlled substance prescriptions filled at outpatient pharmacies in Oregon, regardless of payer, including self-pay.

### Study Sample

We included all adults aged 18 years or older (as of January 1, 2014) with at least 1 valid Oregon zip code between 2015 and 2018. Individuals must also have been continuously enrolled in the APCD member file during at least 1 calendar year in the study period (allowing for 90-day gaps) and must have been in a household of at least 2 members during 1 or more calendar years. We included only years in which individuals were continuously enrolled in the analysis to ensure complete capture of overdose outcomes from the APCD emergency department claims. Persons who would have otherwise met continuous enrollment criteria for the calendar year but died were not excluded in that year. We excluded individuals missing sex (Figure).

**Figure.**
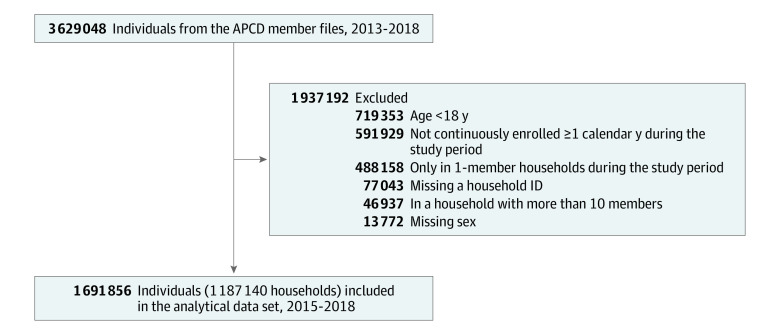
Cohort Flowchart APCD indicates All Payer All Claims Database; ID, identifier.

### Household Assignment

The APCD enrollment files were used to obtain individuals’ addresses; all addresses in the APCD were cleaned, standardized, and geocoded using a process described in detail elsewhere.^12^ For each calendar year, we assigned individuals to a primary address based on where they lived the most during the year. Starting in 2013, we generated household groupers for all individuals in the APCD who shared the same primary address. In a subsequent calendar year, if the same set of individuals continued to live together the household grouper stayed the same, regardless of address. If people left or joined another household in a subsequent calendar year, we generated a new household grouper based on the new set of individuals. Households with more than 10 individuals were excluded from the analyses to avoid inclusion of nursing homes, shelters, correctional institutions, or other large residential group care facilities (0.27% of all addresses in the study period). We included all individuals, including children, in households. If an individual’s insurance was lost or changed to an insurance excluded from the APCD, they would not be placed in a household during those periods.

### Individual Characteristics

We measured age and race and ethnicity as of January 1, 2014. We categorized age into bands: 18 to 24, 25 to 34, 35 to 44, 45 to 54, 55 to 64, 65 to 74, and 75 years and older. We used the Bayesian Imputed Surname Geocoding algorithm to impute race and ethnicity when data were missing from the APCD member file.^14^ Data on race and ethnicity were included to analyze differences in overdose outcomes across race and ethnicity, using data from claims member files. These following categories are largely consistent with how they were reported in claims. The race and ethnicity groups were Asian or Pacific Islander (non-Hispanic), Black (non-Hispanic), Hispanic, White (non-Hispanic), and other (including American Indian and Alaska Native, multiracial, and other race and ethnicity that were not specified by the individual). American Indian and Alaska Native ethnicity was not imputed due to low Bayesian Imputed Surname Geocoding reliability for this population.^15^

For each calendar year in the study period, we used the 2013 National Center for Health Statistics urban-rural classification scheme to classify individuals’ county of residence into metropolitan, nonmetropolitan, or unknown.^16^ We identified the insurance type that patients had for the most days of each year, categorized as commercial, Medicare Advantage, Medicaid, or dual eligibility (Medicare Advantage and Medicaid). Using APCD 2013-2014 medical claims, we identified patient comorbidities using the Elixhauser definitions^17,18^ and pain diagnoses from a validated *International Classification of Diseases, 9th Revision* (*ICD-9*), to *International Statistical Classification of Diseases and Related Health Problems, 10th Revision* (*ICD-10*) crosswalk.^19^ We counted the number of comorbidities and coded them as 0, 1 to 2, or 3 or more.

### Prescription Characteristics

Using the PDMP data set, we identified opioids (including patches) and benzodiazepine prescriptions using the US Food and Drug Administration National Drug Code directory.^20^ We excluded opioid-containing cough and cold formulations including elixirs and combination products containing antitussives, decongestants, antihistamines, and expectorants. We used the Centers for Disease Control and Prevention morphine milligram equivalent (MME) conversion file^21^ to standardize the total opioid dose of each prescription. We kept prescriptions that were not duplicates (had the same date, drug name, quantity, and days’ supply), with a quantity of at least 4 and with MME no greater than the 99th percentile (7200) and had days’ supply between 1 and 90 days. We did not include buprenorphine prescriptions in the total opioid fills for individuals or households but captured them separately, given their unique role as both a pain medicine and a treatment for opioid use disorder.

#### Household-Level Prescription Characteristics

For each individual, we computed prescription characteristics for members of their household (excluding the individual’s prescription characteristics, if present). The household-level prescription characteristics calculated were (1) flags indicating whether anyone else in the household filled a benzodiazepine, buprenorphine, or extended-release opioid formulation; (2) the percentage of household members with an opioid fill; and (3) total opioid dosage (quantity multiplied by the strength and the MME factor, categorized as 0, 1-89, 90-149, 150-299, or ≥300 MME).

We calculated all household prescription characteristics as time-varying monthly measures with a 6-month look back. For instance, to calculate the household’s total MMEs for a month, we added the current month’s total MMEs to the total MMEs the household had in the previous 5 months (6 months total).

#### Individual-Level Prescription Characteristics

To capture personal opioid fills for individuals, we used the same method described for the household level to compute time-varying, monthly personal prescription characteristics with a 6-month look-back. The individual-level prescription characteristics calculated were (1) flags indicating whether the individual filled an opioid, benzodiazepine, buprenorphine, or opioid extended-release formulation; (2) flags indicating fills from 3 or more pharmacies or 3 or more prescribers; (3) flags indicating an overlapping benzodiazepine and opioid fill occurring in the same month; and (4) total opioid dosage (categorized as 0, 1-89, 90-149, 150-299, or ≥300 MME).

#### Household Opioid Availability Indicator

We created a time-varying household availability indicator that updated monthly using a 6-month look back. We based the 4-tiered household opioid availability variable on opioid fills as follows: no opioid fills for the individual or other household members, only the individual filled an opioid, only the other household members filled an opioid, or both the individual and other household members filled an opioid.

### Outcome

For all individuals, we combined fatal and nonfatal opioid overdose data (for both illicit and prescription opioids) to create event indicators for each month of the study period (there was no look-back period for the outcome). Nonfatal opioid overdose events were captured from hospital discharge records and APCD emergency department claims using *ICD-9* (965.00-965.02, 965.09, and E8500-E8502) and *ICD-10* (T40.1-T40.4, and T40.6) codes for opioid poisoning. Fatal opioid overdoses were identified from vital records using *ICD-10* underlying cause-of-death codes (X40-X44, X60-X64, X85, and Y10-Y14) with multiple cause-of-death codes (T40.0, T40.1, T40.2, T40.3, T40.4, and T40.6). Literal text fields in the death record were searched for terms associated with opioid overdose deaths, using an existing tool.^22^

### Statistical Analysis

To assess the association of household opioid availability with increased odds of opioid overdose, we ran population-averaged models, conducted at the person-month level. All models incorporated an R-side residual effect with a first-order autoregressive covariance structure to control for correlations between time points within individuals. Random effects for household were tested but not included in the final models due to convergence issues. The first model used a 4-tiered household opioid availability indicator, with no personal or household member opioid fills as the reference category, to examine the relative association of personal and household opioid fills with opioid overdose. This model was adjusted for individual demographic characteristics, number of comorbidities, and household member count. An additional model examined the association between prescription characteristics and opioid overdose, adjusting for individual demographic characteristics, number of comorbidities, household member count, and the percentage of people in the household with an opioid fill. We calculated adjusted odds ratios (aORs), 95% CIs, and marginal means. All analyses were conducted with SAS Studio, version 9.4 (SAS Institute Inc), and R, version 4.1.3. Analyses were conducted between October 16, 2020, and January 26, 2023.

## Results

A total of 1 691 856 individuals (1 187 140 households; 53.2% women, 46.8% men) were included. Most individuals were of White race (70.7%), lived in metropolitan areas (75.8%), had commercial insurance (51.8%), and had no Elixhauser comorbidities (69.5%) (Table 1). The mean (SD) number of households per person was 1.44 (0.70). The mean (SD) number of study patients per household was 3.14 (1.42, range, 2-10). The percentage of adults who lived with a household member who had at least 1 opioid fill at any point during 2015-2018 was 22.82%. We observed 28 747 (491 fatal and 28 256 nonfatal) opioid overdose events (0.0526 per 100 person-months) of 54 658 632 total person-months.

**Table 1.** Individual Characteristics^a^

Baseline characteristic	No. (%)
Total persons	1 691 856
Total households	1 187 140
Opioid overdoses, No. (rate per 100 patient-months)	28 747 (0.0526)
Age bands, y	
18-24	244 207 (14.4)
25-34	360 271 (21.3)
35-44	315 582 (18.7)
45-54	281 584 (16.6)
55-64	267 673 (15.8)
65-74	202 955 (12.0)
≥75	19 584 (1.2)
Sex	
Female	899 344 (53.2)
Male	792 512 (46.8)
Race and ethnicity	
Asian/Pacific Islander	62 323 (3.7)
Black	47 114 (2.8)
Hispanic	175 940 (10.4)
White	1 195 895 (70.7)
Other^b^	19 878 (1.2)
Unknown	190 706 (11.3)
Urban-rural classification	
Metropolitan	1 281 559 (75.7)
Nonmetropolitan	172 119 (10.2)
Unknown	238 178 (14.1)
Insurance type	
Commercial	876 160 (51.8)
Medicaid	549 543 (32.5)
Medicare Advantage	208 193 (12.3)
Dual^c^	53 167 (3.1)
Other plan	4793 (0.3)
No. of comorbidities^d^	
0	1 175 285 (69.5)
1-2	413 910 (24.5)
≥3	102 661 (6.1)

^a^ Time-varying characteristics, such as insurance type and urban-rural classification, are reported as of the start of the study (January 1, 2015).

^b^ The other race category includes American Indian and Alaska Native individuals, multiracial individuals, and individuals with race and ethnicity that were not specified.

^c^ Dual insured indicates having both Medicare Advantage and Medicaid.

^d^ Comorbidities are flagged using the Elixhauser definitions.

### Association Between Household Opioid Availability and Opioid Overdose

Table 2 presents the association of household opioid availability and overdose. Compared with when neither individuals nor their household members had any opioid fills, individuals’ opioid-related overdose odds increased by 60% when another household member, but not the individual, had recent opioid fills (aOR, 1.60; 95% CI, 1.54-1.66). When the individual, but no other household member, filled opioids in the past 6 months, the odds of overdose were greater (aOR, 5.34; 95% CI, 5.17-5.51), but the odds were highest when both the individual and another household member had opioid fills (aOR, 6.25; 95% CI, 6.09-6.40). Marginal means are reported in eTable 1 in Supplement 1. After adjusting for patient demographic characteristics, patient comorbidities, and household member count, the model-estimated means for opioid overdose were 0.017 per 100 person-months for those with neither household nor person fills, 0.091 per 100 person-months for those with only individual opioid fills, 0.027 per 100 person-months for those with only household opioid fills, and 0.106 per 100 person-months for those with both individual and household fills.

**Table 2.** Association of Household Opioid Availability With Opioid Overdose in an Individual Household Member

Opioid prescription fills in the past 6 mo	aOR (95% CI)^a^
Neither individuals nor household members	1 [Reference]
Only the individual	5.34 (5.17-5.51)
Only household members	1.60 (1.54-1.66)
Both the individual and household members	6.25 (6.09-6.40)

Abbreviation: aOR, adjusted odds ratio.

^a^ Adjusted for patient demographic characteristics, comorbidities, and household member count.

### Association Between Household and Individual Prescription Characteristics and Opioid Overdose

After adjusting for demographic characteristics, household member count, the number of comorbidities, and individual prescription characteristics, household factors associated with increased odds of opioid overdose included total household opioid dosage (1-89 MME: aOR, 1.19; 95% CI, 1.13-1.26; 90-149 MME: aOR, 1.15; 95% CI, 1.08-1.22; 150-299 MME: aOR, 1.13; 95% CI, 1.08-1.19, and ≥300 MME: aOR, 1.21; 95% CI, 1.17-1.25) and a recent benzodiazepine (aOR, 1.12; 95% CI, 1.09-1.16) or buprenorphine (aOR, 1.37; 95% CI, 1.28-1.46) fill by another household member. The percentage of household members with opioid fills was also associated with increased odds of opioid overdose (aOR, 1.03; 95% CI, 1.02-1.04) (Table 3; eTable 2 in Supplement 1 provides marginal means).

**Table 3.** Association of Household and Individual Prescription Characteristics With Opioid Overdose

Prescription characteristics in the past 6 mo	aOR (95% CI)^a^
**Household prescription characteristics**	
MME sum	
0	1 [Reference]
1-89	1.19 (1.13-1.26)
90-149	1.15 (1.08-1.22)
150-299	1.13 (1.08-1.19)
≥300	1.21 (1.17-1.25)
Extended-release formulation	
No	1 [Reference]
Yes	0.94 (0.90-0.99)
No. of buprenorphine fills	
0	1 [Reference]
≥1	1.37 (1.28-1.46)
No. of benzodiazepine fills	
0	1 [Reference]
≥1	1.12 (1.09-1.16)
Percentage of household members with opioid fills (unit change from mean)	1.03 (1.02-1.04)
**Individual prescription characteristics**	
MME sum	
0	1 [Reference]
1-89	2.05 (1.94-2.18)
90-149	2.22 (2.08-2.37)
150-299	2.76 (2.63-2.91)
≥300	3.58 (3.46-3.71)
Extended-release formulation	
No	1 [Reference]
Yes	3.61 (3.46-3.76)
No. of buprenorphine fills	
0	1 [Reference]
≥1	9.28 (8.87-9.70)
No. of benzodiazepine fills	
0	1 [Reference]
≥1	1.80 (1.73-1.88)

Abbreviations: aOR, adjusted odds ratio; MME, morphine milligram equivalent.

^a^ Adjusted for patient demographic characteristics, comorbidities, and household member count.

Increases in total opioid dosage prescribed to individuals were associated with increased odds of opioid overdose (1-89 MME: aOR, 2.05; 95% CI, 1.94-2.18; 90-149 MME: aOR, 2.22; 95% CI, 2.08-2.37; 150-299 MME: aOR, 2.76; 95% CI, 2.63-2.91; and ≥300 MME: aOR, 3.58; 95% CI, 3.46-3.71), as were personal fills of extended-release opioids (aOR, 3.61; 95% CI, 3.46-3.76), buprenorphine fills (aOR, 9.28; 95% CI, 8.87-9.70), and benzodiazepine fills (aOR, 1.80; 95% CI, 1.73-1.88) (Table 3) eTable 2 in Supplement 1 reports marginal means.

## Discussion

This study found that having a household member with a recent opioid prescription fill was associated with increased odds of opioid overdose. Having both a recent personal and household opioid fill was associated with the greatest odds of overdose, followed by having personal fills only. Even without a personal fill, having another household member with a recent opioid fill was associated with a 60% increase in the odds of opioid overdose. These results suggest that a recent opioid fill to a household member is associated with greater odds of opioid overdose, even after accounting for demographic characteristics, comorbidities, and personal fills of opioids and benzodiazepines. Findings from this study are consistent with a growing literature suggesting that household opioid availability is associated with increased odds of opioid-related adverse effects in household members.^10,11,23,24,25,26,27^

While most studies to date have focused on adolescents or young adults in family units who share the same insurance policy number,^10,11,23,28^ our study focused on adults who live at the same address and may not be on the same insurance policy or have familial ties. Using this broader definition of household, our findings are similar to those of previous studies reporting that opioid prescriptions to family members significantly increased youth overdose risk.^11^ Previous researchers have suggested that shared familial genetics, environmental exposures, and norms and behaviors around prescription drug use may account for increased transmission of prescription opioid use through households.^28,29^ Future studies may explore how the association between household opioid availability may differ between familial and nonfamilial households (eg, roommates, nonmarried intimate partners) and how other sociodemographic household factors may play a role.

Several household prescription characteristics were associated with an increased odds of opioid overdose, including the percentage of household members with opioid fills, total household dosages greater than 0, and having 1 or more buprenorphine or benzodiazepine fills. To our knowledge, many of these factors have not been previously explored at the household level, but the findings are consistent with studies suggesting that greater access to opioids or benzodiazepines increases overdose risk.^30,31^ One study examined the role of family member dosage in youth overdose risk, finding only that the highest total dosage to family members (≥600 MME) was associated with increased risk.^11^ In contrast, we found that all total household dosages greater than 0 were associated with increased overdose odds, suggesting the need for further research to understand the role of household dosage in overdose risk. The finding that buprenorphine is associated with increased odds of overdose is difficult to interpret given that we did not stratify by individuals carrying a diagnosis of opioid use disorder. It is possible that buprenorphine is protective in that case, as buprenorphine therapy for opioid use disorder has been found to decrease the risk of opioid-related overdose.^32^

### Limitations

This study has limitations. We used administrative data not originally designed for research and all analyses were retrospective. The probabilistic linkage method may have sometimes resulted in erroneous matches between files. Insurance claims enrollment files were used to obtain individuals’ addresses; if an individual’s insurance was lost or changed to an insurance excluded from the APCD, they would not be placed in a household during those periods. As a result, our data may not have captured all household members, and may have sometimes underestimated opioid prescriptions in a household. Due to the nature of the enrollment data, individuals were assigned to households based on the address where they lived most of the calendar year; if they moved during the year, the household assigned to them may not have been accurate for a portion of the year. Because comorbidities were only measured before the study period and we did not require continuous enrollment during 2013 to 2014, some comorbidities may have been missed. Our analysis examined prescriptions filled during the current month plus the previous 5 months; because we also looked for outcomes during the current month, it is possible that in some cases an outcome may have preceded an exposure. We cannot know from our data how many household opioids have been used, disposed of, or diverted to or from other households during the study period, nor do we know what illicit opioids may be in the household. As a result, we do not have a complete picture of total household opioid availability. The choice of a 6-month look back was arbitrary, but our findings are largely consistent with studies using shorter look-back periods.^11^ In addition, our opioid overdose outcome measure included both illicit and prescription opioids, so we cannot necessarily attribute the overdoses observed in our data to prescribed opioids.

## Conclusions

The results of this large observational cohort study suggest that having recent opioid fills among household members is associated with increased odds of opioid overdose for individuals who live in the same household. These findings underscore the importance of educating patients about the risks of keeping opioids in the household.

## Notes

Supplement 1. **eTable 1.** Marginal Means for Opioid Overdose by Household Opioid Availability **eTable 2.** Marginal Means for Opioid Overdose by Household and Individual Prescription Characteristics

Supplement 2.
Data Sharing Statement
