# Supplementary material for: Association of Household Opioid Availability With Opioid Overdose
Source: JAMA Netw Open. 2023 Mar 17;6(3):e233385. doi: 10.1001/jamanetworkopen.2023.3385 (PMC10024199; doi:10.1001/jamanetworkopen.2023.3385)
Supplement: Supplement 1. — eTable 1. Marginal Means for Opioid Overdose by Household Opioid Availability eTable 2. Marginal Means for Opioid Overdose by Household and Individual Prescription Characteristics [file jamanetwopen-e233385-s001.pdf]

Supplementary Online Content

Hendricks MA, El Ibrahimi S, Ritter GA, et al. Association of household opioid availability with opioid overdose. *JAMA Netw Open*. 2023;6(3):e233385. doi:10.1001/jamanetworkopen.2023.3385

eTable 1. Marginal Means for Opioid Overdose by Household Opioid Availability

eTable 2. Marginal Means for Opioid Overdose by Household and Individual Prescription Characteristics

This supplementary material has been provided by the authors to give readers additional information about their work.

© 2023 Hendricks MA et al. *JAMA Network Open.*

eTable 1. Marginal Means for Opioid Overdose by Household Opioid Availability.

Household opioid availability in the past 6 months	Opioid overdose least square mean per 100 person months (95% CI)
Neither individuals nor household members have opioid fills	0.017 (0.015-0.019)
Only the individual has opioid fills	0.091 (0.081-0.102)
Only household members have opioid fills	0.027 (0.024-0.031)
Both the individual and household members have opioid fills	0. 106 (0.094-0.120)

CI: confidence interval, OR: odds ratio

[a] Adjusted for patient demographics, patient comorbidities and household member count.

© 2023 Hendricks MA et al. *JAMA Network Open.*

eTable 2. Marginal Means for Opioid Overdose by Household and Individual Prescription Characteristics.

Prescription characteristics in the past 6 months	Opioid overdose least square mean per 100 person-months (95% CI)
**Household prescription characteristics**	
**MME sum**	
0	0. 492 (0.435-0.556)
1-89	0. 586 (0.514-0.668)
90-149	0. 563 (0.493-0.643)
150-299	0. 557 (0.490-0.634)
300+	0. 595 (0.525-0.675)
**Extended-release formulation**	
no	0.541 (0.508-0.648)
yes	0.574 (0.477-0.617)
**Number of buprenorphine fills**	
0	0.477 (0.423-0.539)
>=1	0.651 (0.571-0.742)
**Number of benzodiazepine fills**	
0	0.526 (0.465-0.594)
>=1	0.591 (0.522-0.669)
**Individual prescription characteristics**	
**MME sum**	
0	0.261 (0.231-0.295)
1-89	0.535 (0.468-0.610)
90-149	0.578 (0.5045-0.6611)
150-299	0.717 (0.630-0. 816)
300+	0.929 (0.822-1.048)
**Extended-release formulation**	
no	0.2943 (0.260-0.333)
yes	1.053 (0.931-1.19)
**Number of buprenorphine fills**	
0	0.184 (0.162-0.208)
>=1	1.679 (1.484-1.898)
**Number of benzodiazepine fills**	
0	0.416 (0.367-0.471)
>=1	0.747 (0.661-0.845)
**Opioid and benzodiazepine overlap**	
No	0.664 (0.587-0.751)
Yes	0.468 (0.412 -0.530)

CI: confidence interval, MME: morphine milligram equivalent, OR: odds ratio

© 2023 Hendricks MA et al. *JAMA Network Open.*

[a] Adjusted for patient demographics, comorbidities, percentage of household members with opioid fills, and household member count.

© 2023 Hendricks MA et al. *JAMA Network Open.*
