# Supplementary material for: Association of Household Opioid Availability With Opioid Overdose
Source: JAMA Netw Open. 2023 Mar 17;6(3):e233385. doi: 10.1001/jamanetworkopen.2023.3385 (PMC10024199; doi:10.1001/jamanetworkopen.2023.3385)
Supplement: Supplement 2. — Data Sharing Statement [file jamanetwopen-e233385-s002.pdf]

Data Sharing Statement

Hendricks. Association of Household Opioid Availability With Opioid Overdose. *JAMA Netw Open*. Published March 17, 2023. doi:10.1001/jamanetworkopen.2023.3385

Data
Data available: No

Additional Information
Explanation for why data not available: The data used for this study cannot be shared due to state statute.
